# A Systematic Review and Meta-Analysis of Supercapsular Percutaneously Assisted Total Hip Arthroplasty Versus Standard Posterior Approach for Femoral Neck Fracture in Elderly Patients

**DOI:** 10.5435/JAAOSGlobal-D-24-00226

**Published:** 2024-10-21

**Authors:** Zhicheng Pan, Shibo Zhou, Wangxin Liu, Enpeng Gu

**Affiliations:** From the Tianjin University of Traditional Chinese Medicine, Tianjin, China (Dr. Pan and Dr. Liu); the Luoyang Orthopedic-Traumatological Hospital of Henan Province, Henan, China (Dr. Zhou); and the Department of Orthopaedics, The Second Affiliated Hospital of Tianjin University of Traditional Chinese Medicine, Tianjin, China (Dr. Gu).

## Abstract

**Introduction:**

This meta-analysis aimed to evaluate the efficacy and safety of the supercapsular percutaneously assisted total hip arthroplasty (SuperPATH) and the standard posterior approach in hip arthroplasty in treating femoral neck fractures in elderly patients.

**Method:**

A systematic search was conducted for studies from 2012 to December 2022. Meta-analysis was conducted using Review Manager 5.3 on surgical time, intraoperative blood loss, Harris hip scores, and visual analog scale scores.

**Result:**

A total of 26 studies involving 2,236 patients with femoral neck fractures were included. The SuperPATH group performed better than traditional posterior approach group in reducing intraoperative blood loss (in ml), shortening incision length (in cm), length of hospitalization period (in days) and improving Harris Hip score (HHS). The operation time took longer than the traditional posterior approach, with statistically significant differences. The VAS scores at 1 week and 3 months after surgery in the SuperPATH group were lower than those of the traditional posterior approach, with statistically significant differences. There was no statistical significance between the two groups in VAS scores 2 weeks and 1 month after surgery.

**Conclusion:**

The SuperPATH group resulted in better effects in reducing intraoperative blood loss (in ml), shortening incision length (in cm), length of hospitalization period (in days), and improving Harris hip score (HHS), which is conducive to the rapid postoperative recovery of patients.

The femoral neck fracture is a common disease in clinics, accounting for 48% to 54% of hip fractures, and the incidence is increasing yearly. The condition has a particular relationship with the patient's age.^1-3^ With the increase in age, the probability of its occurrence also increases. The femoral neck fracture is known as the “last fracture of life,” which seriously affects patients' quality of life, especially the elderly. Without timely intervention, there will be a variety of complications in the later stage, even life-threatening ones. Hip arthroplasty is one of the most successful surgeries in modern times, which plays an essential role in improving patients' prognosis and quality of life.^4,5^ The standard posterior approach is a familiar and frequently surgical approach for surgeons. The gluteus maximus and external rotator muscles, including the piriformis muscle, are often cut, and the external rotator muscles are reconstructed once the prosthesis is successfully installed. This approach has adverse factors, such as extensive trauma and notable blood loss, which will indirectly lead to a prolonged postoperative recovery period and even joint dislocation and other complications, posing a challenge to doctors and patients.

Rapid rehabilitation after joint surgery has become a research focus with the emergency and progress of minimally invasive technology and the development of the concept of surgical accelerated rehabilitation. Minimally invasive surgical methods such as direct anterior approach arthroplasty and the SuperPATH have emerged. On the premise of ensuring the success of surgery, this kind of surgical method uses small surgical incisions and artificial trauma and gradually attracts the attention of surgeons. The SuperPATH (Supercapsular percutaneously) technology was first reported by Dr Chow in 2011.^6,7^ The SuperPATH, through the piriformis muscle and gluteus minimus muscle space, performs joint arthroplasty on the premise of retaining almost external rotators and joint capsule, ensuring the protection of periarticular muscles, joint capsule, and other soft tissues, which is very conducive to the rapid recovery of patients and in line with the concept of accelerated rehabilitation surgery. It was introduced in China and applied in clinical practice satisfactorily.^8^ Therefore, it is necessary to conduct a meta-analysis to evaluate the efficacy and safety of the SuperPATH in hip arthroplasty for femoral neck fracture and provide more options for surgeons.

## Methods

### Search Strategies

A systematic search for studies from 2012 to December 2022 was done to identify trials comparing the SuperPATH with the standard posterior approach in hip arthroplasty. Relevant researches are searched from Pubmed, the Cochrane Library, Web of Science, Embase, China National Knowledge Infrastructure, Wanfang Database, Chinese Biomedical Literature Database, and the Chongqing VIP Chinese Science and Technology Periodical Database. Keywords were related to femoral neck fractures, hip arthroplasty, and Superpath.

### Inclusion and Exclusion Criteria

#### Inclusion Criteria

(1) Randomized controlled or clinical controlled trials; (2) femoral neck fracture was treated with hip arthroplasty; and (3) the interventions were SuperPATH and standard posterolateral approach.

#### Exclusion Criteria

(1) Case report, surgical technique and learning curve introduction, clinical cost statistics; (2) noncontrolled trials; (3) repeated articles; and (4) outcome measures were incomplete, and The SuperPATH was not observed.

### Data Extraction and Quality Evaluation

#### Data Extraction

The information (including publication year, intervention measures, outcomes, etc.) of the included studies were statistically analyzed by two reviewers, respectively.

#### Risk of Bias Assessment

Cochrane bias risk^9^ includes seven aspects, which are random sequence generation; allocation concealment; blinding of participants and personnel; blinding of outcome; assessment; incomplete outcome data; selective reporting; and other biases. Each section is divided into three levels (low risks, unclear, and high risks).

#### Quality Evaluation

The Modified Jadad^10^ scoring scale was used to evaluate the study quality, including the generation of random sequence generation, blinding of participants and personnel, allocation concealment, loss of follow-up, and withdrawal. Except for withdrawal and loss of follow-up that were classified as “yes” and “no” (marked as 1 and 0, respectively), the other three items all contained three aspects, namely, appropriate, unclear, and inappropriate, which were marked as 2points, 1 point, and 0, respectively. As a result, the total score was seven points, and ≥4 was classified as high-quality studies.

## Outcome Measures

Surgical time: It was defined as the time from the beginning of the skin incision to the suture.

Incision length: It was measured on a graduated scale.

Intraoperative blood loss: Intraoperatively, the total volume of blood aspirated by the suction device is recorded as part of the total blood loss. In addition, all blood-soaked gauzes used during the procedure are weighed, and their initial dry weight is subtracted to determine the volume of blood absorbed. The sum of these two elements constitutes the total intraoperative blood loss.

Length of hospitalization period: It was defined as the time from admission to discharge of the patient.

Harris hip score and visual analog scale (VAS) score were also evaluated.

## Statistical Analysis

Review Manager 5.3 was used to perform the meta-analysis. The odds ratio and 95% confidence interval (95%CI) were used as the effect indicators for the dichotomous variable. The weighted mean difference and 95% confidence interval were used for the continuous variables. When statistical heterogeneity of the study did not exist (I2 < 50%), the fixed-effect model was used. When statistical heterogeneity exists (I2 ≥ 50%), the random-effect model was used. The results of this meta-analysis were shown in the forest plot, and *P* < 0.05 indicated a statistically significant difference.

## Results

### Study Identification and Selection

**Figure 1:**
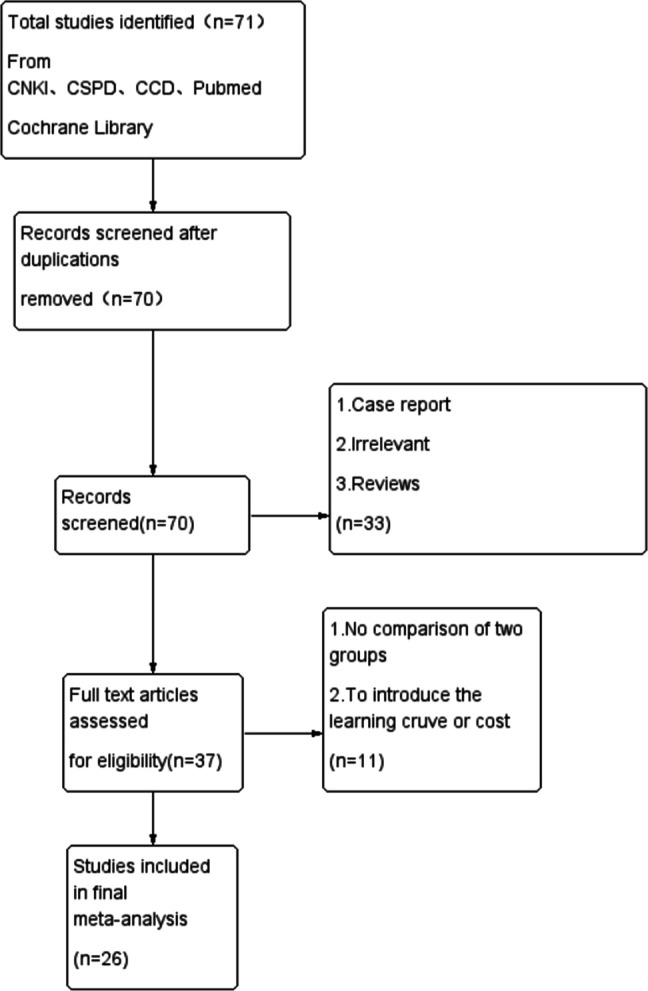
Flow diagram showing literature search and selection.

At the first stage, a total of 71 articles were included. Finally, according to the inclusion and exclusion criteria, 26 studies with 2,236 patients were included (Figure 1 and Table 1).^11-36^

**Table 1:** Characteristics of included studies

Study	PY	Age (years)	Sugery	Corhot Size	Surgical approach	MJS	Outcomes
		S/T		S/T	S/T		
Wang ZJ^11^	2021	71.53 ± 3.76/71.58 ± 3.79	THA	43/42	SP/TP	3	1, 2, 3, 4
Cao LG^12^	2021	68.3 ± 15.34/65.23 ± 14.28	THA	78/60	SP/TP	1	1, 2, 3, 4, 7
Zheng SW^13^	2021	58.86 ± 2.46/55.97 ± 2.62	THA	54/42	SP/TP	1	1, 2, 3, 4, 5, 6, 14, 15, 16,
Chen W^14^	2021	74.30 ± 6.10/75.60 ± 6.90	THA	34/34	SP/TP	1	1, 3, 4, 5
Liu YW^15^	2021	68.27 ± 3.71/68.55 ± 3.40	THA	47/47	SP/TP	3	1, 3, 4, 6, 7, 9
Ling ZL^16^	2020	89.14 ± 3.60/88.95 ± 3.71	THA	50/50	SP/TP	3	1, 2, 3, 4, 7, 18
Hu B^17^	2020	71.70 ± 4.20/72.40 ± 5.10	THA	38/50	SP/TP	1	1, 2, 3, 5, 6, 7, 10
Du B^18^	2020	63.25 ± 5.62/63.23 ± 5.61	THA	48/47	SP/TP	1	5, 6
Gao P^19^	2020	69.26 ± 3.28/68.81 ± 3.45	THA	35/35	SP/TP	1	1, 2, 3, 5, 18, 19, 20
Liu XC^20^	2019	63.18 ± 5.36/65.98 ± 7.59	THA	60/67	SP/TP	1	1, 2, 3, 4, 5, 6, 7
Chen C^21^	2019	69.20 ± 6.50/68.50 ± 6.10	THA	28/25	SP/TP	1	1, 2, 3, 4
Li G^22^	2019	71.3 ± 10.75/70.28 ± 7.76	THA	40/40	SP/TP	1	1, 2, 3, 4, 6
Wang^23^	2019	69.03 ± 3.01/70.13 ± 3.35	THA	55/55	SP/TP	3	1, 2, 3, 4, 5, 6
Jia^24^	2019	78.10 ± 2.30/79.50 ± 2.60	HHA	50/50	SP/TP	2	1, 2, 3, 6, 7
Wu GH^25^	2018	63.60 ± 3.10/64.40 ± 3.30	HHA	14/25	SP/TP	1	1, 2, 3, 5, 7
Li MZ^26^	2021	75.26 ± 4.35/76.46 ± 5.14	HHA	41/41	SP/TP	3	1, 3, 5, 6
Dai GH^27^	2019	69.9/70.3	HHA	61/67	SP/TP	3	1, 2, 3, 4, 5, 6, 7, 18
Zhang JP^28^	2017	78/77	HHA	22/64	SP/TP	1	1, 2, 3, 6, 7, 18
Wu LH^29^	2017	—	HHA	20/20	SP/TP	4	1, 2, 3, 4, 6, 7
Xu GF^30^	2018	70.81 ± 6.08/71.02 ± 5.96	HHA	46/46	SP/TP	3	1, 2, 3, 4, 6, 7, 18
Ding BC^31^	2018	81.18 ± 5.93/80.76 ± 5.57	HHA	50/50	SP/TP	3	1, 2, 3, 5, 20
Xia LZ^32^	2018	81.00 ± 4.57/80.66 ± 4.26	HHA	30/32	SP/TP	2	1, 2, 3, 6, 16
Huang JJ^33^	2021	73.22 ± 4.15/74.09 ± 4.23	HHA	35/35	SP/TP	3	1, 2, 3, 4, 5, 18
Ding YT^34^	2018	68.90 ± 6.08/68.51 ± 6.14	HHA	42/41	SP/TP	3	1, 5, 7, 18
Wang XC^35^	2021	67.84 ± 7.35/67.15 ± 6.58	HHA	50/50	SP/TP	2	1, 2, 3, 4, 6, 7, 9, 18
Zhao L^36^	2019	81.50 ± 5.20/82.80 ± 6.30	HHA	25/25	SP/TP	3	1, 2, 3, 4, 6

HHA = hip hemiarthroplasty, PY = published year, S = SuperPATH Group, T = standard posterior approach group, SP = SuperPATH, TP = standard posterior approach, THA = total hip arthroplasty.

1 = surgical time, 2 = intraoperative blood loss, 3 = incision length, 4 = length of hospitalization period, 5 = VAS: visual analog scale, 6 = Harris hip score, 7 = postoperative drainage volume, 8 = visual analog score in 24 hours, 9 = SF-36:the MOS item short from health survey, 10 = CK: creatine kinase, 11 = acetabular abduction angle, 12 = acetabular anteversion angle, 13 = EQ-5D: EuroQol five dimensions questionnaire, 14 = ESR: erythrocyte sedimentation rate, 15 = CRP: C-reactive protein, 16 = Hb: hemoglobin, 17 = creatine kinase, 18 = weight-bearing, 19 = TUG: Timed Up and Go.

### Characteristics of the Studies

Table 1 gives an overview of the main characteristics of the 26 included studies published between 2017 and 2021, involving 2,236 patients. Of these, 1,140 patients underwent surgery through the SuperPATH (644 patients with total hip arthroplasty and 496 patients with hemiarthroplasty) and 1,096 patients through the standard posterior approach (660 patients with THA and 436 patients with hemiarthroplasty). Two studies were published in English (41,42), nine in Chinese only (45), and the other 15 in Chinese with English abstracts. All patients were diagnosed with a femoral neck fracture.

### Included Studies Quality Bias Risk Results and Jadad Score

**Figure 2:**
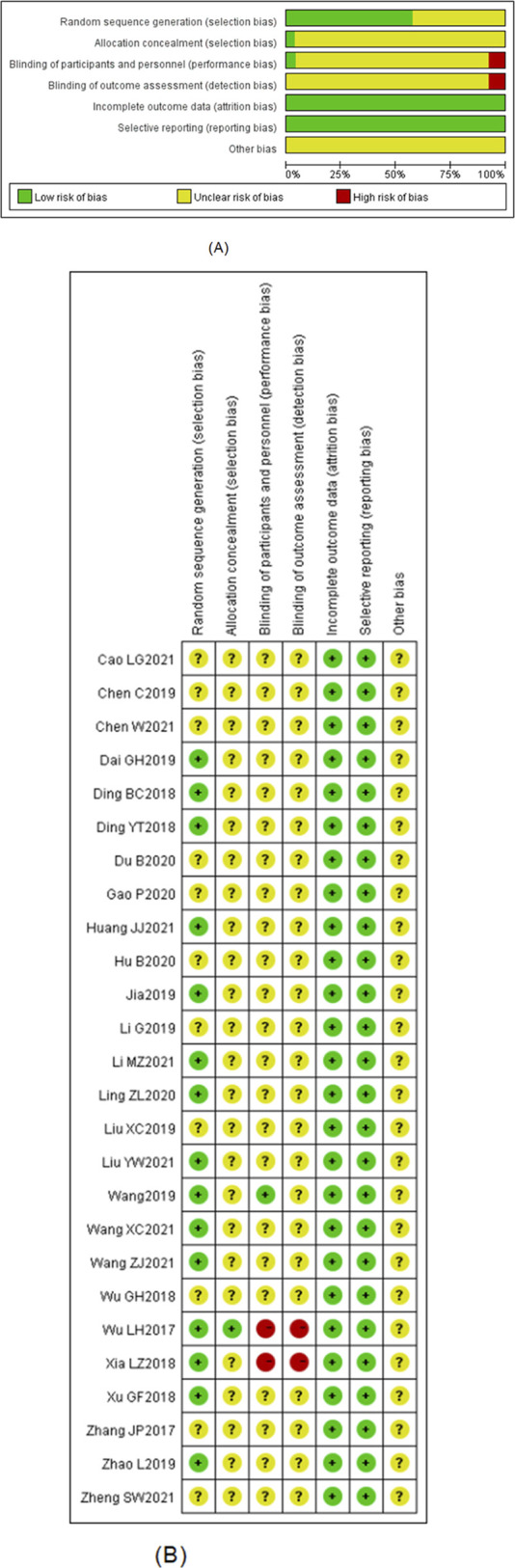
Graph showing **A,** risk of bias and (**B**) risk of bias summary.

A total of 12 studies mentioned the random number method, and three studies mentioned the randomness. Still, they did not mention specific methods; 11 studies did not mention randomness, one study mentioned allocation hiding, 24 studies did not describe whether a blind method was used or not, and two studies indicated that an open and nonblind method was used. The data of the included studies were relatively complete, and no cases of loss of visit or withdrawal were found. No selective reports were carried out in the studies, and other risks were unknown. Jadad score: one high-quality study was there, and the Jadad score of the remaining studies was between one and three points, with most three points. The overall quality of the studies was average (Figures 2, A, B).

## Outcomes

### Surgical Time

**Figure 3:**
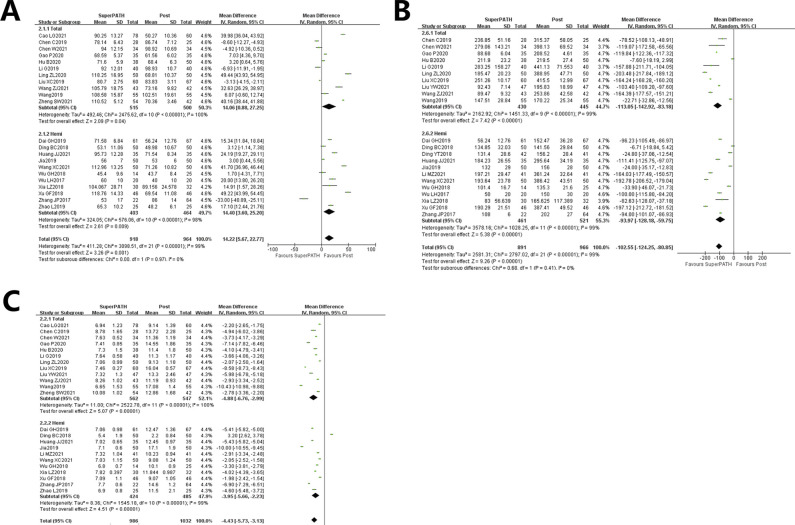
Forest plot of comparison of surgical time, intraoperative blood loss, and incision length.

Data from 22 studies (19-22,24-25,27-33,35-41,43-44) on 1882 patients (including 918 in the SuperPATH group and 964 in the standard posterior approach group) were pooled for analyzing the surgical time. Compared with the standard posterior approach, the surgical time of the SuperPATH group was longer with statistically significant differences [I2 = 99%, MD = 14.22, 95%CI = (5.67, 22.77), Z = 3.26, *P* = 0.001; Figure 3; post: standard posterior approach].

#### Subgroup analysis:

The surgical time analysis showed that THA (19-22,24-25,27-31) and hemiarthroplasty (32-33, 35-41,43-44) were statistically significant. The surgical time of the SuperPATH group was longer than that of the standard posterior approach group: total hip arthroplasty: [MD = 14.06, 95%CI = (0.88, 27.25), Z = 2.09, *P* = 0.04] and hemiarthroplasty: [MD = 14.40, 95%CI = (3.60,25.20), Z = 2.61, *P* = 0.009; Figure 3].

### Intraoperative Blood Loss

Data from 22 studies (19,22-25,27-43) analyzing the intraoperative blood loss were pooled on 1857 patients (including 918 patients in the SuperPATH group and 966 patients in the standard posterior approach group). Compared with the standard posterior approach group, the SuperPATH group had less intraoperative blood loss with statistically significant differences [I2 = 99%. MD = −102.55, 95%CI = (−124.25, −80.85), Z = 9.26, *P* < 0.00001; Figure 3B; post: standard posterior approach].

#### Subgroup Analysis

Subgroup analysis of intraoperative blood loss shows that both THA (19,22-25,27-31) and hemiarthroplasty (32-43) of the SuperPATH group were less than that of the standard posterior approach, with statistically significant differences: Total hip Arthroplasty [MD = −113.05, 95%CI= (−142.92,−83.18), Z = 7.42, *P* < 0.00001] and hemiarthroplasty [MD = −93.97, 95%CI=(−128.18, −59.75), Z = 5.38, *P* < 0.00001; Figure 3B].

### Incision Length

Data on 2018 patients (including 986 patients in the SuperPATH group and 1032 patients in the standard posterior approach group) were pooled from 23 studies (19-25,27-36,38-41,43-44) analyzing the incision length. Compared with the standard posterior approach group, the incision length of the SuperPATH group was shorter than that of the standard posterior approach group with statistically significant differences [I2 = 99%, MD = −102.55, 95%CI = (−124.25, −80.85), Z = 9.26, *P* < 0.00001; Figure 3C; post: standard posterior approach].

#### Subgroup analysis

Subgroup analysis of incision length shows that both THA (19-25,27-31) and hemiarthroplasty (32-36,38-41,43-44) of the SuperPATH group were shorter than that of the standard posterior approach with statistically significant differences: Total Hip Arthroplasty [MD = −4.88, 95%CI = (−6.76,2.99), Z = 5.07, *P* < 0.00001] and hemiarthroplasty [MD = −3.95, 95%CI = (−5.66, −2.23), Z = 4.51, *P* < 0.00001; Figure 3C].

### Length of Hospitalization Period

**Figure 4:**
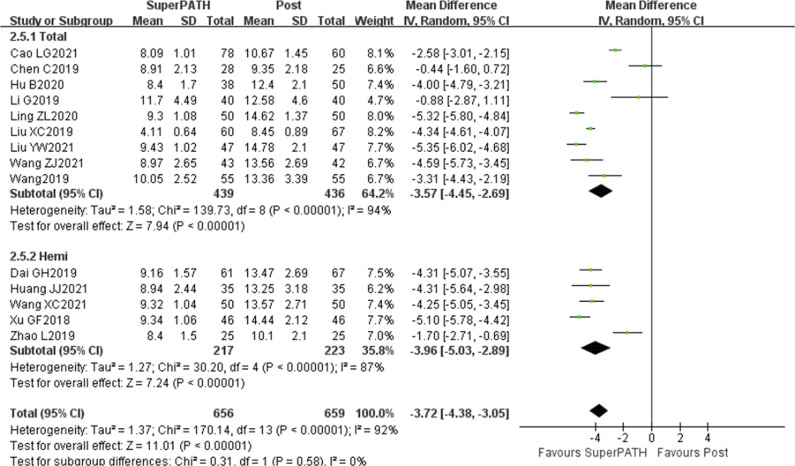
Forest plot of comparison of length of hospitalization period.

Data from 14 studies (19-20,23-25,28-31,35,38,41,43-44) on the length of hospitalization period was pooled on 1315 patients (including 656 patients in the SuperPATH group and 659 patients in the standard posterior approach group). Compared with the standard posterior approach group, the length of hospitalization period of the SuperPATH group was shorter than that of the standard posterior approach group, with statistically significant differences [MD = −3.72, 95%CI = (−4.38, −3.05), Z = 11.01, *P* < 0.00001; Figure 4; post: standard posterior approach].

#### Subgroup analysis

Subgroup analysis of the length of hospitalization period shows that both THA (19-20,23-25,28-31) and hemiarthroplasty (35,38,41,43-44) in the SuperPATH group were shorter than those in the standard posterior approach with statistically significant differences: Total Hip Arthroplasty [MD = −3.57, 95%CI= (−4.45, −2.69), Z = 7.94, *P* < 0.00001] and hemiarthroplasty [MD = −3.72, 95%CI = (−5.03, −2.89), Z = 7.24, *P* < 0.00001; Figure 4].

### Visual Analog Scale Score

A total of 1,007 patients (including 501 patients in the SuperPATH group and 506 patients in the standard posterior approach group) were pooled from 10 studies (21-22,25-27,31-32,34-35,41), analyzing the VAS score.

**Figure 5:**
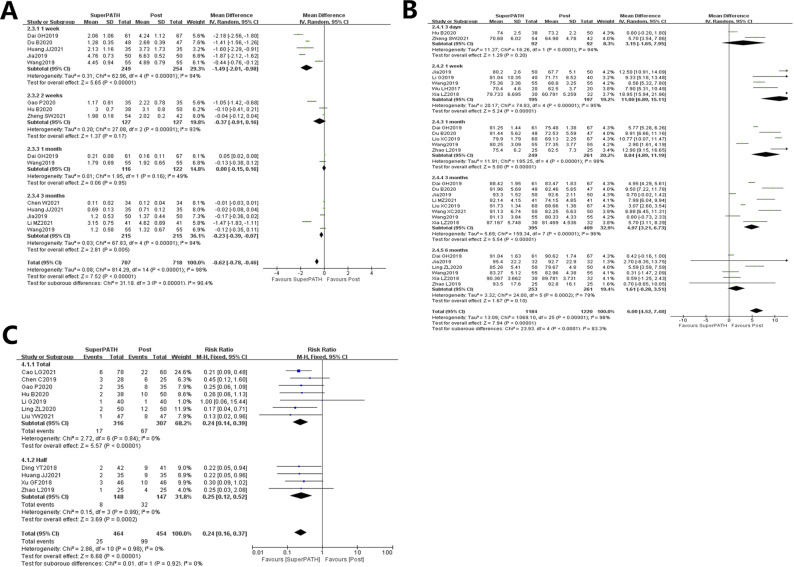
Forest plot of comparison of VAS score, Harris hip score, and security.

Compared with the standard posterior approach group, the SuperPATH group demonstrated lower VAS scores with statistically significant differences [MD = −0.62, 95%CI = (−0.78, −0.46), I2 = 98%, Z = 7.52, P ＜ 0.00001; Figure 5A; post: standard posterior approach].

#### Subgroup analysis

VAS score at one week and three months postoperatively show that both THA and hemiarthroplasty of the SuperPATH group were lower than the standard posterior approach with statistically significant differences [1 week: MD = −1.49, 95%CI=(−2.01, −0.98), I2 = 94%,Z = 5.65, *P* < 0.00001]; [3 months: MD = 0.23, 95% CI = (0.39, 0.07), Z = 2.81, *P* = 0.005; Figure 5A].

Data from five studies (26,31-32,35,41) analyzing the VAS 1 week postoperatively on 503 patients (including 249 patients using the SuperPATH and 254 patients using the standard posterior approach) were pooled. The results showed that the VAS of the SuperPATH group was lower than that of the standard posterior approach, with statistically significant differences [MD = −1.49, 95%CI = (−2.01,−0.98), I2 = 94%, Z = 5.65, *P* < 0.00001; Figure 5A].

Data from three studies (21,25,27) analyzing the VAS 2 weeks after the surgery were pooled to include 254 patients (127 with the SuperPATH group and 127 with the standard posterior approach). No difference was found between the two groups concerning the VAS 2 weeks postoperatively [MD = −0.37, 95%CI = (−0.91,0.16), I2 = 93%, Z = 1.37, *P* = 0.17; Figure 5A].

Data from two studies (31,35) analyzing the VAS 1 month postoperatively were pooled to include 238 patients (116 with the SuperPATH group and 122 with the standard posterior approach). No difference was found between the two groups concerning the VAS 1 month postoperatively [MD = 0, 95%CI = (−0.15, 0.16, I2 = 49%, Z = 0.06, *P* = 0.95; Figure 5A).

Data on 430 patients (including 215 patients with the SuperPATH group and 215 patients with the standard posterior approach) were pooled from five studies (22,31-32,34,41) analyzing the VAS 3 months postoperatively. The results showed that the VAS of the SuperPATH group was lower than that of the standard posterior approach, with statistically significant differences [MD = −0.23, 95%CI = (−0.39,−0.07), I2 = 94%, Z = 2.81, *P* = 0.005; Figure 5A].

### Harris Hip Score

Data from 14 studies (21,24-26,28,30-32,34-35,37,40,43-44) analyzing the Harris hip score were pooled on 1,429 patients (including 687 patients in the SuperPATH group and 742 patients in the standard posterior approach group). Compared with the standard posterior approach group, the HHS score of the SuperPATH group was lower than the standard posterior approach group with statistically significant differences [MD = 6.00, 95%CI = (4.52, 7.48), I2 = 98%, Z = 7.94, P ＜ 0.00001; Figure 5B; post: standard posterior approach].

Subgroup analysis of HHS at one week, one month, and three months postoperatively showed that the HHS of the SuperPATH group was higher than the standard posterior approach with statistically significant differences [1 week: MD = −1.49, 95%CI = (6.89, 15.11), Z = 5.24, P ＜ 0.00001]; [1 month: MD = 8.04, 95%CI=(4.89,11.19), Z = 5.00,P ＜ 0.00001]; [3 months: MD = 4.97, 95% CI=(3.21, 6.73), Z = 5.54, P ＜ 0.00001; Figure 5B).

Data from two studies (21,25) analyzing the HHS 3 days postoperatively were pooled to include 184 patients (92 with the SuperPATH group and 92 with the standard posterior approach). No difference was observed between the two groups concerning the HHS 3 days postoperatively [MD = 3.15, 95%CI = (−1.65, 7.95), I2 = 94%, *P* = 0.20; Figure 5B).

Data from five studies (30-31,37,40) analyzing the HHS 1 week postoperatively were pooled to include 392 patients (195 with the SuperPATH group and 197 with the standard posterior approach). The results showed that the HHS of the SuperPATH group was higher than that of the standard posterior approach, with statistically significant differences [MD = −1.49, 95%CI = (6.89, 15.11), I2 = 95%, Z = 5.24, P ＜ 0.00001; Figure 5B].

Data from five studies (26,28,31,35,44) analyzing the HHS 1 month postoperatively were pooled on 510 patients (including 249 patients using the SuperPATH and 261 patients using the standard posterior approach). The results showed that the HHS of the SuperPATH group was higher than that of the standard posterior approach, with statistically significant differences [1 month: MD = 8.04, 95%CI = (4.89, 11.19), I2 = 98%, Z = 5.00, P ＜ 0.00001; Figure 5B];

Data from eight studies (26,28,31-32,34-35,40,43) analyzing the HHS 3 months postoperatively were pooled on 804 patients (including 395 patients using the SuperPATH and 409 patients using the standard posterior approach). The results showed that the HHS of the SuperPATH group was higher than that of the standard posterior approach, with statistically significant differences [3 months: MD = 4.97, 95%CI = (3.21, 6.73), I2 = 96%, Z = 5.54, P ＜ 0.00001; Figure 5B].

Data from six studies (24,31-32,35,40,44) analyzing the HHS 6 months postoperatively were pooled on 514 patients (including 253 patients using the SuperPATH and 261 patients using the standard posterior approach). No difference was observed between the two groups concerning the HHS 6 months postoperatively [MD = 1.61, 95% CI = (−0.28,3.51), I2 = 79%, Z = 1.67, *P* = 0.10; Figure 5B].

### Security

Data from 11 studies (20,23-25,27,29-30,38,41-42,44) analyzing postoperative complications were pooled on 918 patients (including 464 in the SuperPATH group and 454 in the standard posterior approach group). Compared with the standard posterior approach group, the postoperative complications of the SuperPATH group was lower than that of the standard posterior approach group with statistically significant differences [RR = 0.24, 95%CI = (0.16, 0.37), *P* < 0.00001; Figure 5C; post: standard posterior approach].

Subgroup analysis of postoperative complications shows that both THA (20,23-25,27,29-30) and hemiarthroplasty (38,41-42,44) of the SuperPATH group could reduce the postoperative complications (including dislocation, infection, venous thrombosis, and bedsore), compared with the standard posterior approach group with statistically significant differences: Total Hip Arthroplasty [MD = 0.24, 95%CI = (0.14, 0.39), Z = 5.57, P ＜ 0.00001] and hemiarthroplasty [MD = 0.25, 95%CI = (0.12, 0.52), Z = 3.69, *P* = 0.0002; Figure 5C].

### Sensitive Analysis

After excluding the included RCTs one by one, the final results were not reversed regarding the surgical time, incision length, length of hospitalization period, intraoperative blood loss, HHS, and postoperative complications with statistically significant differences, suggesting that the above results were relatively stable and reliable. After eliminating Chen Wei (22), Li Meiji (34), and Huang Jijia (41) one by one, the VAS score results were reversed, indicating that the VAS score results were unstable.

## Publication Bias

Among the included studies, two mentioned the total effective rate, and four described the excellent and reasonable rates of postoperative Harris score for hip joints. Few related studies were found, and the potential publication bias was not detected through the funnel plot. Regarding the quality and sample size of the included studies, a possibility of publication bias was found in the meta-analysis results. If more studies mentioned the total effective rate, publication bias could be analyzed.

## Discussion

The occurrence of femoral neck fractures increases with age, especially in elderly patients. This is closely related to the deterioration of the physical function of older people, relatively slow reactions, and osteoporosis.^37^ Hip arthroplasty for treating femoral neck fractures in elderly patients has become one of the most successful surgical methods since modern times. Several surgical approaches have been developed. The standard posterior approach is sufficiently exposed to facilitate the operator's surgery. However, it is necessary to split the gluteus maximus muscle, cut off the piriformis muscle and other external rotator muscles, destroy the joint capsule, and damage the soft tissue around the joint, which is not conducive to the postoperative recovery of patients, resulting in prolonged recovery time, and even dislocation of the joint and other serious complications.^38,39^ With the progress of minimally invasive technology and the rise of accelerated rehabilitation in joint surgery, minimally invasive hip arthroplasty has increasingly become a need. Under the premise of protecting soft tissue as much as possible, the surgery can better maintain the physiological structure of the human body and reduce complications. The SuperPATH enters the joint capsule through the piriformis muscle and gluteus minimus muscle space. It can effectively protect the periarticular piriformis muscle and other external rotator muscles and fully protect the surrounding soft tissues. Smaller incisions and better soft-tissue protection also provide an excellent anatomical basis for early postoperative recovery, which is conducive to rapid postoperative recovery of patients.

## Conclusion

The SuperPATH for hip arthroplasty in treating femoral neck fractures in elderly patients had satisfactory effects and was generally safe and effective. This surgical approach is superior to the standard posterior approach in reducing intraoperative blood loss, shortening incision length, length of hospitalization period, reducing VAS score, increasing HHS, and reducing postoperative complications, which is conducive to rapid postoperative recovery of patients, and the results are stable and reliable. According to the findings, although the surgical time was longer than the standard posterior approach, the postoperative complications did not increase due to the prolonged surgical time. On the premise that the patient's soft tissues are protected, timely functional exercise can help avoid postoperative complications, which also confirms the safety of the surgical method.

Disadvantages: Chinese studies were mainly included in this meta-analysis, although English studies mainly introduced surgical techniques, learning curves, and statistically related costs. According to the Modified Jadad score, the number of high-quality studies included at this time was limited, and the studies did not consider whether the patients had severe underlying diseases in the past. It is unknown whether there is an effect on the length of the hospitalization period, functional scores, and other outcomes. More high-quality RCTs are needed to confirm this result further.
